# The Aswan Rheumatic heart disease reGIstry: rationale and preliminary results of the ARGI database

**DOI:** 10.3389/fcvm.2023.1230965

**Published:** 2023-09-18

**Authors:** Susy Kotit, Magdi H. Yacoub

**Affiliations:** ^1^ Aswan Heart Centre, Aswan, Egypt; ^2^ Heart Science Centre, National Heart and Lung Institute, Imperial College London, London, United Kingdom

**Keywords:** Rheumatic heart disease, Egypt, Aswan, database, registry

## Abstract

**Background:**

Rheumatic Heart Disease (RHD) remains a major cause of valvular heart disease related mortality and morbidity in low- and middle-income countries, with significant variation in characteristics and course of the disease across different regions. However, despite the high disease burden, there is sparse region-specific data on demographics, disease characteristics and course in treated and untreated patients to guide policy.

**Methods:**

The ARGI database is a hospital-based registry in a tertiary referral national centre (Aswan Heart Centre, AHC) in which all patients with the diagnosis of RHD are being included. The mode of presentation, including baseline clinical and echocardiographic characteristics (as well as other imaging modalities), biomarkers and genetics are being documented. Treatment modalities and adherence to treatment is being recorded and patients are followed up regularly every 6 and/or 12 months, or more frequently if needed.

**Discussion:**

This study shows for the first time an in-depth analysis of the severity and phenotype of disease in Egyptian patients presenting with RHD as well as the progression with time and provides a platform for further comparisons of regional differences in these details as well as their causes. The ARGI database will be of help in achieving the objectives of the Cairo Accord aiming at eradication of RF and RHD.

## 1. Introduction and rationale for the study

Rheumatic heart disease (RHD) is the most common cause of acquired heart disease in children and young adults globally (1, 2) and remains a major health care problem causing significant morbidity and mortality at all ages. On global scale, RHD caused 305,651 deaths in 2019 and led to nearly 10.7 million (9.2–12.1) DALYs lost (3). In 2019, there were 2.8 million new cases and 40.5 million prevalent cases of RHD, representing 49.7% and 70.5% increases since 1990, respectively.

The characteristics and course of the disease vary across different regions (4–9). However, there is sparse data on RHD demographics, disease characteristics, age distribution, course of the disease, adverse events, the need and timing of medical interventions, long term outcomes, and mortality (4, 10, 11). To date, there is no similar data from Egypt (12, 13).

Development of databases for accurate data on epidemiology and natural disease history is vital for the prevention and control of RHD, as recommended in the Cairo Accord aiming at disease eradication (15). Comparing the results of different regional registries and the main causative factors, including the influence of genetics and epigenetics should help the global efforts to eradicate RHD (16, 17).

The Aswan Heart Centre (AHC) is a high volume tertiary referral centre with over 45,000 patients seen in outpatient clinics yearly, in which around one-quarter of the workload is related to RHD. The number of patients requiring interventions for RHD is increasing at AHC, and while there are other centres dealing with the disease, there is a pressing need to increase access to surgery for a population of 105 million individuals.

Furthermore, in spite of current medical and surgical treatment, adverse events remain significant (4, 14). Of particular interest remains the thrombosis of prosthetic valves which has been highlighted in a previous publication from AHC (14) but requires continuous efforts.

Our objective is to establish a RHD registry (Aswan Rheumatic heart disease registry, ARGI) to provide disease and regional specific data which could enhance the understanding of the global and regional epidemiology of RHD.

## 2. Methods

### 2.1. Study design

The ARGI database is a hospital-based registry in which all patients from all over Egypt with the diagnosis of RHD are being included.

#### 2.1.1. Objectives

The ARGI database was designed to address the following aims:

(1) Mode of presentation
(2) Type of treatment (and adherence, including penicillin prophylaxis)
(3) The outcome over time

#### 2.1.2. Patient cohort and study eligibility

All consecutive patients with a primary diagnosis of RHD (clinical or echocardiographic) seen at the out-patient clinics (OPD) and inpatient facilities at AHC are eligible to participate (Algorithm 1).

**ALGORITHM 1:**
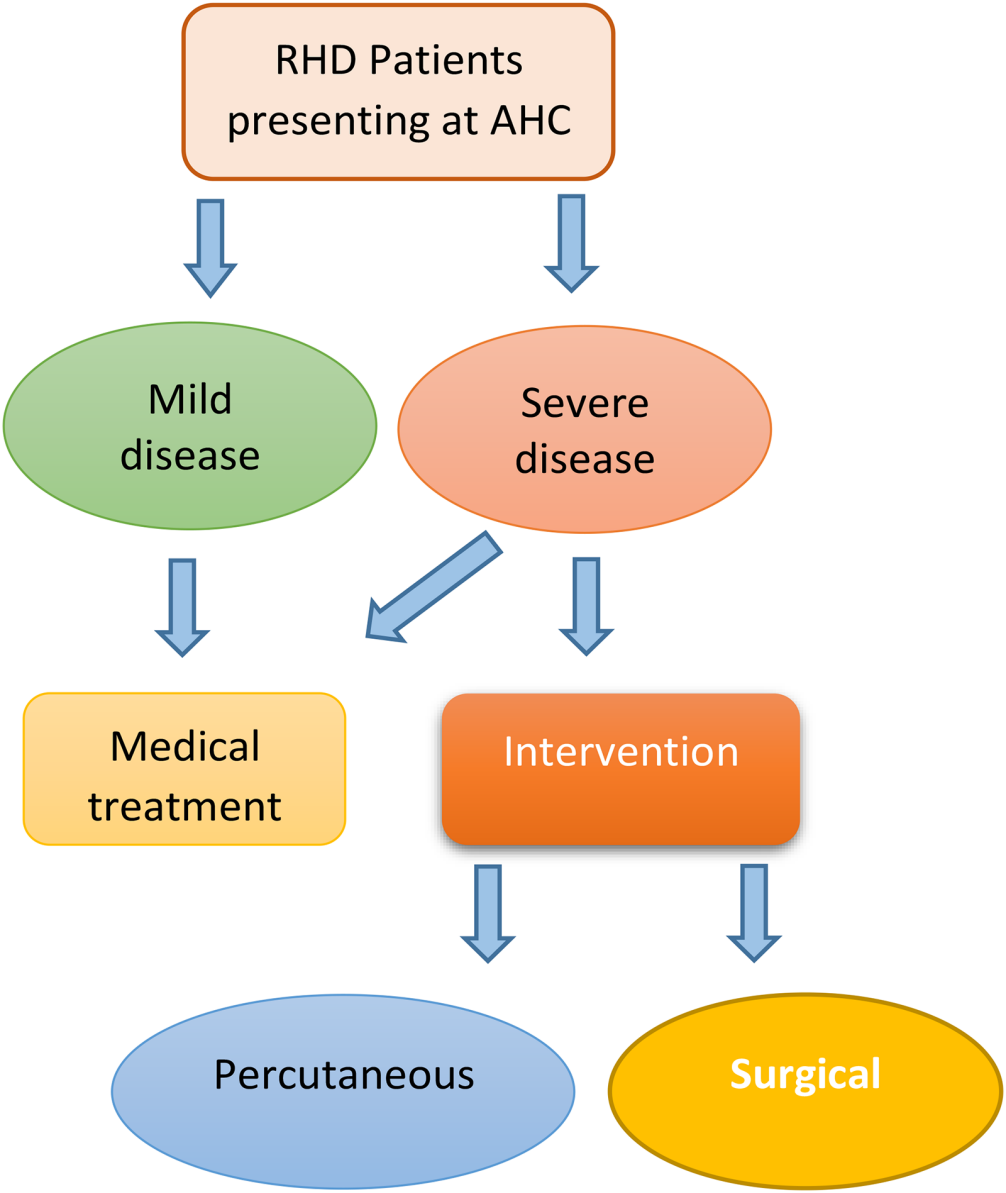
Patient flow chart.

#### 2.1.3. Plan of investigation

(1) Mode of presentation
  (a) Demographic characteristics (age and gender, etc), history of ARF
  (b) Valves affected (the pattern and severity of valvular involvement)
  (c) Previous interventions
(2) Type of treatment at the AHC and patient adherence
  (a) Pharmacologic treatments, antibiotic prophylaxis, oral anticoagulation
  (b) Intervention (percutaneous, surgical)
(3) Monitoring Rheumatic activity
  (a) BioBank (blood samples taken at regular intervals from all patients (6-months))
  (b) Collection of all surgically explanted tissues from repaired or replaced valves
(4) Monitoring outcome over time
  (a) Adverse events
  (b) Progression of cardiac disease (progression valvular disease, heart failure, etc)

### 2.2. Data collection

#### 2.2.1. Mode of presentation

Demographic information (age, gender, social class, geographic distribution, familial incidence, history of ARF) and clinical data (BP, heart rate, height, weight, medical history, co-morbidities, and previous interventions) are being collected at intervals of 6 months.

Echocardiography is performed for the analysis of the pattern and severity of valvular involvement at presentation and at each follow-up visit, classified using the current World Heart Federation (WHF) criteria (Supplementary Table S1) (18). Cardiac echocardiographic studies are stored online on a specialized platform. All images are reviewed by experienced cardiologists and the heart team.

MRI, CT and cardiac catheterization are performed when indicated.

#### 2.2.2 . Genetics and biomarkers

Blood samples are being taken for biomarkers and DNA extraction. Following separation of the blood into serum and cell components as well as extraction of DNA, the samples are stored in the biobank for future analysis.

#### 2.2.3. Treatment modalities

(A) Pharmacologic treatments and adherence are being documented, particularly secondary antibiotic prophylaxis, oral anticoagulation and anti-arrhythmic therapy
(B) Percutaneous (Balloon mitral valvuloplasty)
(C) Surgical interventions

#### 2.2.4. Follow-up

Quality of life, adverse events (Supplementary Table S2), exercise capacity, LV and RV function, pulmonary hypertension and progression of valve disease are being assessed during 6 monthly follow-up visits. All-cause mortality is being monitored.

### 2.3. Study management

Management of the database is based at the AHC. The principal investigators (PI's) are responsible for the management of the registry, overseeing data collection and quality assurance. The PI's have been responsible for the development of the CRFs, consent forms, patient information sheets, and management algorithms, in addition to the development and maintenance of the web-based database.

#### 2.3.1. Ethics

The study has been approved by the Magdi Yacoub Foundation-AHC Research Ethics Committee (AHC-REC) in accordance with current practices, a consent form is obtained for specimen collection, storage and analysis. Detailed information sheets have been developed and are provided to each participant. All invasive investigations performed are according to prevailing standard of care guidelines.

### 2.4. Status of the study

**Figure 1:**
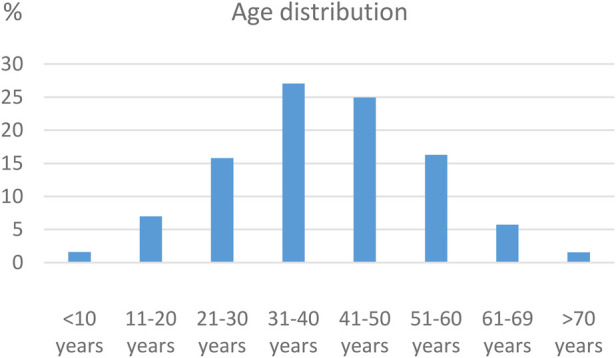
Age distribution at presentation (baseline).

**Figure 2:**
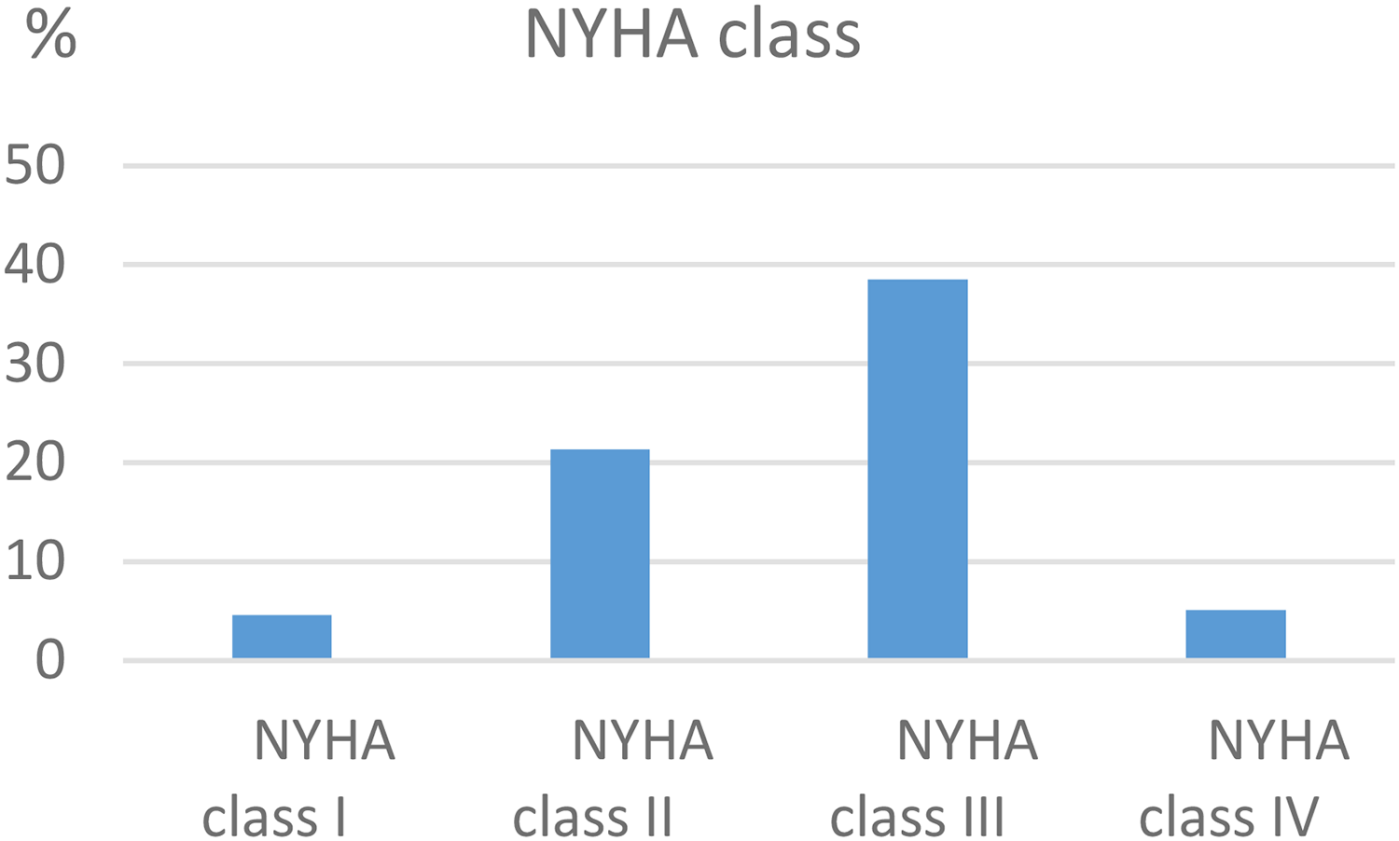
Distribution of NYHA class at presentation.

A total of 2,510 consecutive patients with clinical and echocardiographic RHD have been enrolled in the ARGI database since March 2009. The age at the time of the first visit ranged from 3 to 86 years (40.11 ± 13.86) (Figure 1). The majority was female (*n* = 1,695, 67.5%). Only 123 patients (4.9%) had history of RF of which 23 (0.92%) were on secondary penicillin prophylaxis. Symptoms were present in 75.8% (*n* = 1,902) of the patients, with dyspnea present in 69.6% (*n* = 1,746) (Figure 2).

**Figure 3:**
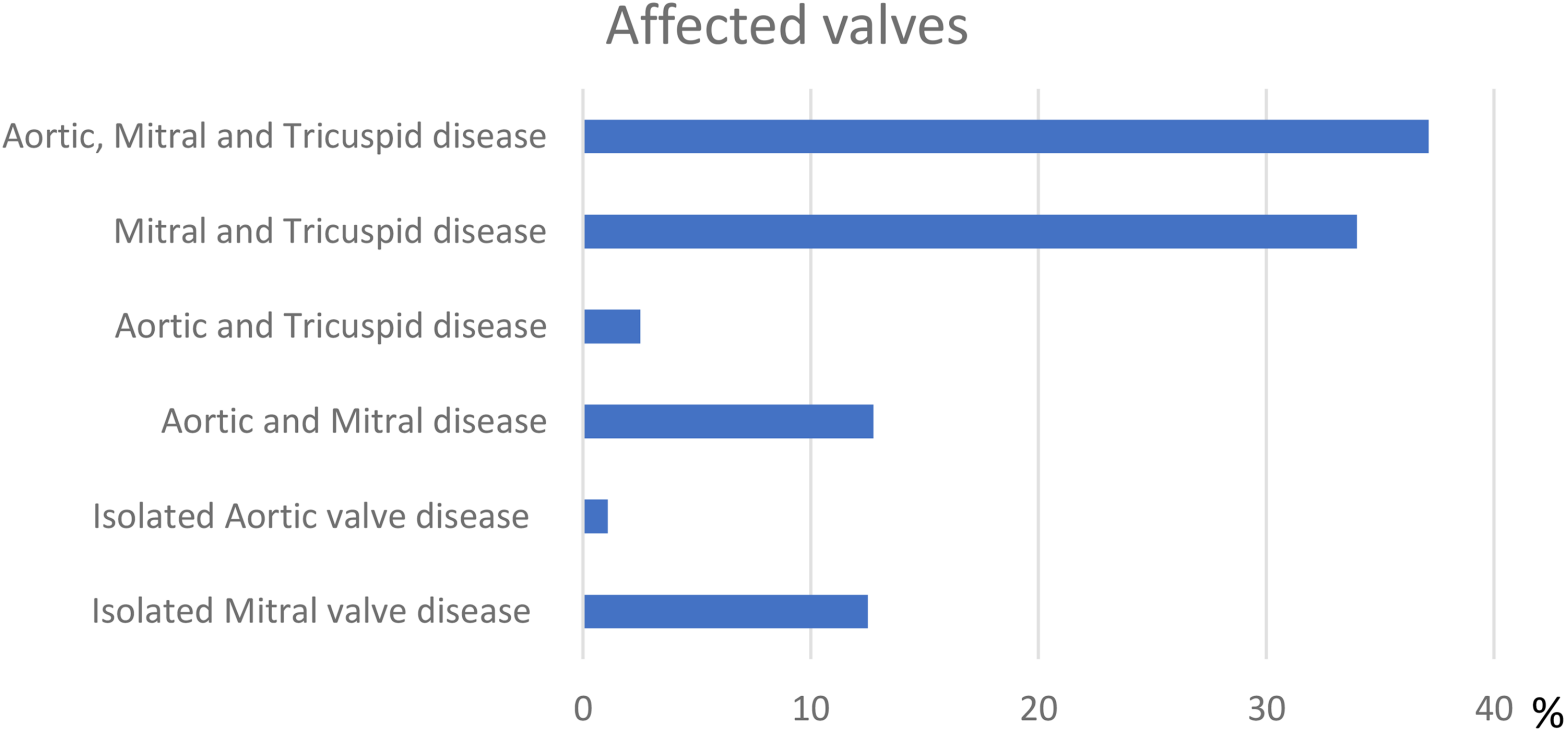
Valve affection.

At enrolment, the majority (*n* = 2,287, 91.1%) of patients had moderate-to-severe valvular disease (Table 1 and Figure 3) complicated by atrial fibrillation (AF) (*n* = 723, 28.8%), cerebrovascular events (*n* = 146, 5.8%), infective endocarditis (*n* = 16, 0.63%) and thrombosis of valve prosthesis (*n* = 12, 0.47%). Previous cardiac intervention prior to the first visit to our centre was reported in 19.4% of the patients (*n* = 487). The age at the time of the first intervention ranged from 4 to 68 (28.21 ± 11.47). The most common first intervention reported was percutaneous balloon mitral valvoplasty (BMV) (*n* = 232, 47.6%).

**Table 1:** Dominant valve pathology.

Dominant valve pathology	*n=*	%
Mitral stenosis	1,034	41.195
Mitral regurgitation	903	35.976
Aortic stenosis	146	5.817
Aortic regurgitation	423	16.853
Tricuspid regurgitation	4	0.159

During regular 6 monthly follow-up at our centre (*n* = 1,795) for periods ranging from 6 to 142 months (39.46 ± 34.18) (Table 2) a total of 1,303 (72.6%) patients underwent cardiac procedures (Table 3). The age at the time of the intervention ranged from 5 to 79 years (35.64 ± 12.63) at an average of 49.4 months since first presentation (range: 0–140 months, SD: 37.3 months).

**Table 2:** Patient characteristics at the time of last follow-up.

Patient characteristics at the time of last follow-up	*n = *1,795 (range)	%
Follow-up time (months)	6–142	39.46 ± 34.18
Gender (female) %	1,202	67.0
Age (years)	5–90	44.15 ± 15.7
Symptoms	1,136	63.3
NYHA class		
NYHA class I	523	29.1
NYHA class II	294	16.4
NYHA class III	153	8.5
NYHA class IV	16	0.9

**Table 3:** Interventions.

Type of intervention	*n*	%
BMV	286	21.95
MV repair	317	24.33
AV repair	12	0.92
AV + MV repair	19	1.46
Ross procedure	22	1.69
+MV repair	11	0.84
Valve replacement-AV		
Prosthetic	39	2.99
+MV repair	14	1.07
+MVR	40	3.07
+TVR bio-prosthesis	1	0.08
+MVR + TVR	1	0.08
+MVR + TVR (bio)	2	0.15
Bio-prosthesis	87	6.68
+MV repair	62	4.76
+MVR	21	1.61
+MVR bio-prosthesis	2	0.15
+MVR (bio) +TVR (bio)	1	0.08
Valve replacement-MV		0.00
Prosthetic	258	19.80
+AV repair	7	0.54
+CABG	3	0.23
+TVR	6	0.46
+TVR bio-prosthesis	6	0.46
Bio-prosthesis	69	5.30
+TVR bio-prosthesis	1	0.08
Valve replacement-TV	2	0.15
Other	14	1.07

Cerebrovascular events (*n* = 83, 4.6%), LA thrombus (*n* = 20, 1.1%), infective endocarditis (*n* = 13, 0.7%) and thrombosis of valve prosthesis (*n* = 18, 1%) occurred during follow-up. Patient mortality was 16% (*n* = 287) during the study period (39.46 ± 34.18 months), of which 162 (56.4%) patients had history of intervention.

Penicillin prophylaxis rate was 34.7% (*n* = 623), however, only 12.9% of these patients followed a correct penicillin prophylaxis schedule.

### 2.5. Limitations

The ARGI database deals with late chronic disease and initiatives to diagnose earlier phases of disease including ARF (15, 19, 20) as well as population studies (21) are essential.

## 3. Discussion

The preliminary results show for the first time an in-depth analysis of the severity and phenotype of disease in Egyptian patients presenting with RHD as well as the progression with time. The ARGI database provides a platform for further comparisons of regional differences in these details as well as their causes.

In addition, with the increasing numbers of patients recruited, the ARGI study will be combined and compared to similar studies from Egypt (13, 22, 23).

It is hoped that the ARGI study will be of help in achieving the objectives of the Cairo Accord aiming at eradication of RF and RHD.

## Data Availability

The datasets presented in this study can be found in online repositories. The names of the repository/repositories and accession number(s) are: https://redcap.ahc-research.com/redcap/ with accession number: PID 117. Further enquiries can be directed to the corresponding author(s).
