# Supplementary material for: The Aswan Rheumatic heart disease reGIstry: rationale and preliminary results of the ARGI database
Source: Front Cardiovasc Med. 2023 Sep 18;10:1230965. doi: 10.3389/fcvm.2023.1230965 (PMC10545855; doi:10.3389/fcvm.2023.1230965)
Supplement: Supplementary file 1 [file Table1.docx]

**Supplementary Material**

**Table S1**

The WHF echocardiographic diagnostic criteria of RHD

|  | Morphological features | Pathological regurgitation  (All four doppler criteria must be met) |
| --- | --- | --- |
| Mitral valve | - AMVL thickening ≥3 mm (age-specific) - Chordal thickening - Restricted leaflet motion - Excessive leaflet tip motion during systole | - Seen in two views - In at least one view, jet length ≥2 cm - Velocity ≥3 m/s for one complete envelope - Pan-systolic jet in at least one envelope |
| Aortic valve | - Irregular or focal thickening - Coaptation defect - Restricted leaflet motion - Prolapse | - Seen in two views - In at least one view, jet length ≥1 cm - Velocity ≥3 m/s in early diastole - Pan-systolic jet in at least one envelope |
| Definite RHD (either A, B, C, D, or E)   1. Pathological MR and at least two morphological features of RHD of the MV 2. MS mean gradient ≥4 mmHg 3. Borderline disease of both the AV and MV (in individuals ≤20 years) 4. Pathological AR and at least two morphological features of RHD of the Av (in individuals <35 years) 5. Pathological AR and at least two morphological features of RHD of the MV (in individuals >20 years) | | |
| Borderline RHD (either A, B, or C)   1. At least two morphological features of RHD of the MV without pathological MR or MS 2. Pathological MR 3. Pathological AR | | |
| AMVL: anterior mitral leaflet; AR: aortic regurgitation; MR: mitral regurgitation; MS: mitral stenosis (adapted from [18]) | | |

[18] Reményi B, Wilson N, Steer A, Ferreira B, Kado J, Kumar K, et al. World Heart Federation criteria for echocardiographic diagnosis of rheumatic heart disease--an evidence-based guideline. Nat Rev Cardiol. 2012 Feb 28;9(5):297-309. doi: 10.1038/nrcardio.2012.7. PMID: 22371105; PMCID: PMC5523449.
