# Supplementary material for: The Aswan Rheumatic heart disease reGIstry: rationale and preliminary results of the ARGI database
Source: Front Cardiovasc Med. 2023 Sep 18;10:1230965. doi: 10.3389/fcvm.2023.1230965 (PMC10545855; doi:10.3389/fcvm.2023.1230965)
Supplement: Supplementary file 2 [file Table2.docx]

**Table S2**

| Endpoint/ Outcome definitions | |
| --- | --- |
| Outcome | **Definition** |
| Death | Death due to any cause |
| Congestive heart failure | Any 2 of the 3 following criteria: (i) signs (rales, increased jugular venous pressure or ankle edema) or symptoms (dyspnea on exertion or at rest, orthopnea, nocturnal paroxysmal dyspnea, or ankle edema) of congestive heart failure, (ii)radiologic signs of pulmonary congestion, and (iii) treatment with diuretics |
| Stroke | Diagnosis of stroke by a physician based on sudden onset of neurologic deficit consistent with ischemia/infarction of a vascular territory, lasting ≥24h, with or without confirmation by neuroimaging |
| Transient ischemic attack | Deficits diagnosed by a physician lasting <24 h |
| Non-CNS systemic embolism | Diagnosed clinically in patients with loss of arterial pulse and/or evidence of end-organ ischemia (eg,ischemic limb pain, gangrene, etc) with or without confirmation by Doppler studies or arteriography |
| Major bleeding | Bleeding that (i) is fatal, (ii) involves a critical site (intracranial, retroperitoneal, intraspinal, intra ocular, pericardial, or intra-articular), or (iii) leads to a reduction in hemoglobin level ≥2 g/dL, or requires transfusion of ≥2 units of whole blood or packed red cells |
| New-onset AF or flutter | Diagnosis of AF or flutter with or without ECG evidence |
| ARF | Diagnosed by the current WHO criteria [1] |
| Infective endocarditis | Diagnosed by the modified Duke criteria [2] |
| Valve surgery | Performance of any valve repair, or replacement of valve with a tissue, or mechanical prosthesis |
| Percutaneous valvular interventions | Percutaneous balloon dilatation of stenosed mitral, aortic tricuspid, or pulmonary valves |
| Prosthetic valve thrombosis | Recent onset (≤2 wk) symptoms of valve dysfunction (dyspnea, angina, or congestive heart failure) accompanied by new onset of restricted valve leaflet motion on cine-fluoroscopy with or without increased valve gradients on Doppler echocardiography |
| CNS indicates Central nervous system; WHO, World Health Organization; ARF, Acute Rheumatic Fever; AF, atrial fibrillation | |

1. Gewitz, MH, Baltimore, RS, Tani LY, Sable CA, et al. Revision of the Jones Criteria for the Diagnosis of Acute Rheumatic Fever in the Era of Doppler Echocardiography: A Scientific Statement From the American Heart Association. Circulation, 2015 May 19; 131(20): 1806- 1818. [doi.org/10.1161/CIR.0000000000000205](https://doi.org/10.1161/CIR.0000000000000205)

# 2. Durack DT, Lukes AS, Bright DK. New criteria for diagnosis of infective endocarditis:

# utilization of specific echocardiographic findings. Duke Endocarditis Service. Am J

# Med. 1994 Mar;96(3):200-9. doi: 10.1016/0002-9343(94)90143-0. PMID: 8154507.
